# Open-Label Sulforaphane Trial in FMR1 Premutation Carriers with Fragile-X-Associated Tremor and Ataxia Syndrome (FXTAS)

**DOI:** 10.3390/cells12242773

**Published:** 2023-12-05

**Authors:** Ellery Santos, Courtney Clark, Hazel Maridith B. Biag, Si Jie Tang, Kyoungmi Kim, Matthew D. Ponzini, Andrea Schneider, Cecilia Giulivi, Federica Alice Maria Montanaro, Jesse Tran-Emilia Gipe, Jacquelyn Dayton, Jamie L. Randol, Pamela J. Yao, Apostolos Manolopoulos, Dimitrios Kapogiannis, Ye Hyun Hwang, Paul Hagerman, Randi Hagerman, Flora Tassone

**Affiliations:** 1 Medical Investigation of Neurodevelopmental Disorders (MIND) Institute, University of California Davis Health, Sacramento, CA 95817, USAanschneider@ucdavis.edu (A.S.); rjhagerman@ucdavis.edu (R.H.);; 2 Department of Pediatrics, School of Medicine, University of California, Davis, CA 95817, USA; 3 Division of Biostatistics, Department of Public Health Sciences, University of California, Davis, CA 95616, USA; 4 Department of Molecular Biosciences, School of Veterinary Medicine, University of California, Davis, CA 95616, USA; 5 Child and Adolescent Neuropsychiatry Unit, Department of Neuroscience, Bambino Gesù Children’s Hospital, IRCCS, 00165 Rome, Italy; federica.montanaro@opbg.net; 6 Department of Education, Psychology, Communication, University of Bari Aldo Moro, 70121 Bari, Italy; 7 Department of Biochemistry and Molecular Medicine, School of Medicine, University of California, Davis, CA 95616, USA; 8 Laboratory of Clinical Investigation, Intramural Research Program, National Institute on Aging, National Institutes of Health, Baltimore, MD 212241, USAapostolos.manolopoulos@nih.gov (A.M.); kapogiannisd@mail.nih.gov (D.K.)

**Keywords:** FXTAS, FMR1, neurodegeneration, sulforaphane

## Abstract

Fragile X (FMR1) premutation is a common mutation that affects about 1 in 200 females and 1 in 450 males and can lead to the development of fragile-X-associated tremor/ataxia syndrome (FXTAS). Although there is no targeted, proven treatment for FXTAS, research suggests that sulforaphane, an antioxidant present in cruciferous vegetables, can enhance mitochondrial function and maintain redox balance in the dermal fibroblasts of individuals with FXTAS, potentially leading to improved cognitive function. In a 24-week open-label trial involving 15 adults aged 60–88 with FXTAS, 11 participants successfully completed the study, demonstrating the safety and tolerability of sulforaphane. Clinical outcomes and biomarkers were measured to elucidate the effects of sulforaphane. While there were nominal improvements in multiple clinical measures, they were not significantly different after correction for multiple comparisons. PBMC energetic measures showed that the level of citrate synthase was higher after sulforaphane treatment, resulting in lower ATP production. The ratio of complex I to complex II showed positive correlations with the MoCA and BDS scores. Several mitochondrial biomarkers showed increased activity and quantity and were correlated with clinical improvements.

## 1. Introduction

The fragile X (*FMR1*) premutation (55 to 200 CGG repeats) is a common mutation occurring in approximately 1 in 200 females and 1 in 450 males [1,2]. Individuals with this premutation often develop fragile-X-associated tremor/ataxia syndrome (FXTAS) with age, one of the most common monogenic neurodegenerative disorders. The syndrome, first reported in 2001, includes an intention tremor, cerebellar ataxia, cognitive decline, neuropathy, and brain changes detected by magnetic resonance imaging (MRI) [3,4]. The MRI changes include broadly distributed brain atrophy and white matter disease at specific locations, such as the middle cerebellar peduncles (MCPs), corpus callosum, insula, and periventricular areas [5]. FXTAS can occur in up to 75% of males with the premutation by the eighth decade of life [6] and in approximately 16% of females [7,8], with clinical signs typically emerging in the early 60s [9]. Thus, FXTAS is a relatively common aging-related syndrome that is underdiagnosed and understudied [10]. There is no specific, effective treatment for FXTAS, although a variety of medications may improve psychiatric issues (i.e., selective serotonin reuptake inhibitors; SSRIs) or the severity of tremor [11] (beta blockers or primidone).

Both patients with FXTAS and animal models of the *FMR1* premutation demonstrate cellular calcium dysregulation, mitochondrial dysfunction, and oxidative stress. One of the critical features of mitochondrial dysfunction in FXTAS is impaired oxidative phosphorylation (OXPHOS) [1,2,3]. The levels of mitochondrial complex I, III, and IV proteins, as well as the activity of these complexes, are reduced in the brains of FXTAS patients, with the cause of death being uncertain [12]. Studies have shown that individuals with FXTAS have mitochondrial DNA (mtDNA) mutations and increased mtDNA deletions in their brains, indicating impaired mitochondrial function. This leads to deficiencies in energy production and increased oxidative stress [13].

Furthermore, a study of FXTAS skin fibroblasts showed that mitochondrial respiratory capacity is compromised and the mitochondria morphology is altered, with the presence of fragmented and swollen mitochondria [14]. This suggests that the regulation of mitochondrial dynamics is also disrupted in FXTAS, which can further contribute to mitochondria dysfunction [13]. As neurons are highly dependent on the aerobic energy provided by mitochondria, oxidative stress and mitochondrial dysfunction can reduce neuronal viability [15]. Despite these various reports indicating mitochondrial abnormalities in individuals with FXTAS, it is unclear whether mitochondrial dysfunction represents a cause or a contributor to the pathogenesis of the condition.

Sulforaphane is an antioxidant, anti-inflammatory, and mitochondrial-protective agent that has been studied in several animal models and humans with neurodegenerative disorders [16]. Sulforaphane is found in cruciform vegetables, seeds, and plants, including broccoli, cauliflower, and Brussels sprouts. Previous studies have shown that sulforaphane activates and up-regulates the *NFE2L2* (nuclear factor erythroid 2-related factor 2) pathway by promoting the dissociation of NFE2L2 from its inhibitor, Kelch-like ECH-associated protein 1 (KEAP1) [17]. NFE2L2, encoded by the *NRF2*/*NFE2L2* gene, is a component of the cellular defense mechanism that regulates the expression of antioxidant and detoxification enzymes [18]. NFE2L2 exerts its effect by promoting the transcription of genes required to control oxidative stress damage while restoring redox homeostasis [19]. In addition to increasing mitochondrial biogenesis, NFE2L2 also plays a role in inhibiting autophagy and mitophagy and inhibiting mitochondrial fission [20].

The effects of sulforaphane were studied in fibroblasts obtained from eight males with FXTAS and four control lines without the premutation by analyzing the proteome pre- and post-treatment with sulforaphane [21]. The study showed a significant up-regulation of NFE2L2-mediated proteins essential for redox homeostasis, improved mitochondrial function, and lowered immune dysfunction in fibroblasts from FXTAS-affected patients at relatively high stages. This observation presents a compelling basis for evaluating both the safety and potential in vivo effectiveness of sulforaphane in FXTAS patients. These findings prompted us to investigate the effects of sulforaphane in an open-label 6-month trial on biomarkers of mitochondrial dysfunction and clinical measures in the treatment of those with FXTAS.

## 2. Materials and Methods

### 2.1. Study Protocol

This study was conducted at the Fragile X Treatment and Research Center at the MIND Institute at the University of California, Davis Health, with the approval of the UC Davis Institutional Review Board. It was registered at ClinialTrials.gov (NCT05233579). All participants gave signed, informed consent before enrollment. All patients had an *FMR1* premutation allele documented via *FMR1* DNA testing and a diagnosis of FXTAS (see Table 1 and Table 2 for the diagnostic criteria) [11,22]. A total of 15 participants were enrolled in the trial from July 2021 to October 2022.

Eligibility criteria included ages between 50–85 years, documentation of the premutation with *FMR1* DNA testing, and a previous diagnosis of probable FXTAS or definite FXTAS and FXTAS stages between 2 and 5 [4,23]. Critical exclusion criteria were as follows: hypersensitivity to cruciform vegetables; severe renal failure (GFR was <60 mL/min/1.73 m^2^); significant substance abuse (6 or more symptoms of substance use disorder); any terminal disease; and MRI contraindications such as ferrous metal in any part of the body. Participants continued their regular medications during the treatment study.

**Figure 1:**
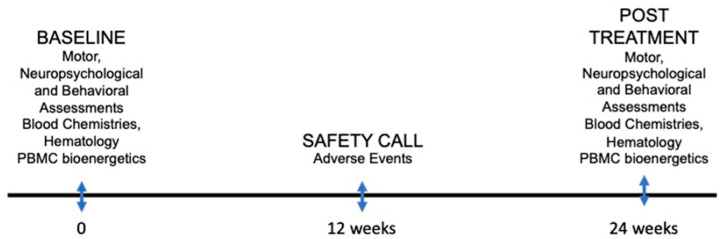
Open-label sulforaphane trial design.

The study consisted of 3 visits: baseline/start of sulforaphane treatment, 12 weeks, and 24 weeks (Figure 1). Phone calls were made monthly to review adverse events (AEs) or problems with the medication. The 12-week visit was conducted as a phone follow-up visit. Medical history, physical examination including vital signs, adverse events reporting, and SCL-90R, ADLs, and CGI-I were performed at baseline and 24 weeks. Hematology (CBC and differential), chemistry panel (CHEM12), and biomarkers were also obtained.

Administration of sulforaphane and protocol schedule: All enrolled participants were provided capsules of Avmacol^®^, a sulforaphane-producing dietary supplement that contains glucoraphanin, the precursor to sulforaphane, and active myrosinase enzyme that catalyzes the reaction of glucoraphanin to sulforaphane. The supplements were administered orally, starting with 1 tablet in the morning with breakfast. Then, every other day, the dose was increased by 1 tablet to 6 tablets/day in the morning. If the maximum amount was not tolerated because of bloating, gas, or indigestion, the highest dose was continued throughout the 6-month study. Capsules were dispensed to participants in sealed bottles provided by Nutrimax and kept at room temperature. Participants stopped the supplementation after their 24-week visit.

Safety and tolerance: Adverse event monitoring and documentation by duration, severity, and relatedness were performed at the 12-week follow-up visit and at 24 weeks (end of study). Participants were requested to keep daily medication diaries, which they utilized to record dosage information, timing of administration, and any side effects, such as bloating, constipation, or diarrhea. The study drug was stopped if it was no longer tolerated at the starting dose or if laboratory values were above study eligibility rules (which did not happen). Compliance was ascertained by residual pill counts and medication diaries.

Primary and secondary outcomes: Our main aim and focus was on evaluating alterations in molecular biomarkers associated with mitochondrial dysfunction and ROS (reactive oxygen species) on sulforaphane. Additionally, we aimed to assess the biomarker correlations in conjunction with clinical measures and explore whether any enhancements in clinical outcomes could be observed as secondary objectives.

Neuropsychological, psychopathological, and behavioral assessments: The assessments were conducted by staff, including a psychologist and physician, and were overseen by the PI (RJH). All had had extensive experience with patients with FXTAS. Except for the intellectual assessment (IQ) that was carried out only at baseline, all the other tests listed and described below were administered at baseline (T0) and after six months (T2).

Cambridge neuropsychological test automated battery (CANTAB) [24]: Originally designed in the 1980s, the CANTAB is one of the most automated cognitive batteries currently used. It effectively differentiates between normal populations and individuals with clinical conditions such as dementia and cognitive impairment [25]. The battery includes the following subtests: intra–extra dimension set shift, spatial working memory strategy, spatial working memory test, within-search errors spatial span, rapid visual information processing, paired-associates learning test (PAL), spatial recognition memory, verbal recognition memory, and pattern recognition memory. The strength of the battery is that it is language-independent and culturally neutral.

Symptom checklist-90-revised (SCL-90-R): This test battery aims to evaluate a broad range of psychological problems in people aged 13 or older, and it was used at baseline and during the last two weeks before testing [26]. The 90-item questionnaire measures nine primary symptom constructs: somatization, obsessive compulsive disorder, interpersonal sensitivity, depression, anxiety, hostility, phobic anxiety, paranoid ideation, and psychoticism. Each item has the five following response categories: 0 = not at all, 1 = little, 2 = some, 3 = very, and 4 = severe. The global severity index can be used as a summary of the test.

Behavioral dyscontrol scale, 2nd edition (BDS-II): The behavioral dyscontrol scale (BDS) [27] is a 9-item measure of dynamic motor behavior, alphanumeric sequencing, and insight, initially validated for geriatric individuals. Subsequently, the BDS-II scoring system [28] was created to measure executive functioning in younger and high-functioning individuals. In the BDS-II, a total score ranging from 0 to 27 is derived from the same nine BDS items that can be scored from 0 to 3. Items 1 and 2 require the participant to alternatingly tap once with one hand and twice with the other; item 3 is a go–no-go task; in item 4, the respondent taps his hand once when the examiner taps twice, and vice versa; items 5 and 6 measure motor procedural learning; in item 7 the participant is asked to mimic the examiner’s movements; item 8 is an auditory alphanumeric sequencing task, similar to trail making part B; finally, item 9 evaluates the patient’s insight regarding performance on the test.

Montreal cognitive assessment (MoCA): The MoCA test is a short screening instrument usually administered to people with cognitive loss [29]. It assesses different domains, which include attention and concentration, memory, orientation, language, visuo-constructional skills, conceptual thinking, calculations, and executive functions. A maximum of 30 points are attainable, with a score of 26 being used as a cut-off score between normal and pathological.

### 2.2. Biomarkers

*FMR1* genotyping and expression levels: Genomic DNA was isolated from peripheral blood samples (3 mL) using a Gentra Puregene Blood Kit (Qiagen, Valencia, CA, USA). CGG repeat size was obtained using PCR and Southern blot analysis, as previously described [30,31]. Capillary electrophoresis (CE) was used to visualize and size the PCR products. The methylation status of the *FMR1* alleles was assessed by Southern blotting, as detailed in [4,5,32]. *FMR1* mRNA levels were measured using qRT-PCR using Assays-On-Demand (Applied Biosystems, Foster City, CA, USA) and custom TaqMan primers and probe assays, as reported in Tassone et al. (2000) [33].

FMRP expression levels: FMRP was quantified via the time-resolved fluorescence resonance energy transfer (TR-FRET) method using a Cisbio Human FMRP assay kit (Cisbio US, Bedford, MA, USA) according to the manufacturer’s recommendations except for the following: (i) Protease inhibitors were added to frozen peripheral blood mononuclear cells (PBMCs) during thawing. (ii) Cells were lysed in Cisbio lysis buffer supplemented with Benzonase (MilliporeSigma, Burlington, MA, USA) in the presence of MgCl_2_ to reduce viscoelasticity. Fluorescent antibody conjugates were incubated with cell lysates at room temperature for 18 h. A control fibroblast fiducial line was used to generate a standard curve to interpolate the percentage change in fluorescence (ΔF%), as performed by Kim et al. 2019 [34]. A four-factor fit was used for ⊗F% > 65, while a linear fit was used for ⊗ F% < 65 to allow for the interpolation of negative replicate values. Interpolated FMRP values were then corrected to total protein loaded, as determined by a BCA Protein Assay (Thermo Fisher Scientific, Rockford, IL, USA). Finally, the relative FMRP level was calculated by normalizing the historical mean of samples with control alleles. 

Plasma neuron-derived extracellular vesicles (NDEV) isolation and mitochondrial measures: EDTA plasma aliquots were received and processed blindly by investigators at the National Institute on Aging, Baltimore, MD. NDEVs were isolated by immunoaffinity capture targeting L1 Cell Adhesion Molecule (L1CAM), a transmembrane neuronal protein sorted to EVs. The methodology followed has been described previously in detail [35,36]. Briefly, 250 μL of plasma was defibrinated with 100 μL of thrombin followed by the addition of 150 μL of Dulbecco’s PBS-1X (DPBS), supplemented with protease/phosphatase inhibitors, and sedimented at 3000× *g* for 15 min at room temperature (RT). The supernatant was transferred to a sterile 1.5 mL microtube, and particles were sedimented by incubation with 126 μL of ExoQuick™ followed by centrifugation at 1500× *g* for 30 min at RT. Pelleted crude total EVs were resuspended by overnight gentle rotation mixing at 4 °C in 350 μL of ultra-pure distilled water supplemented with protease/phosphatase inhibitors. Resuspended crude total EVs were then incubated for 30 min at RT with 4 μg of biotinylated anti-human L1CAM antibody. EV–antibody complexes were incubated with 25 μL of washed Pierce™ Streptavidin Plus UltraLink™ Resin for 30 min at RT. After centrifugation at 600× *g* for 10 min at 4 °C and removal of unbound EVs and soluble proteins in the supernatant, NDEVs were eluted with 100 μL of 0.1 M glycine, followed promptly by pH normalization. Beads were sedimented by centrifugation at 4000× *g* for 10 min at 4 °C, and the supernatant containing immunoprecipitated NDEVs was transferred to a sterile tube.

Using a commercial assay, we measured mitochondrial complex IV in NDEVs (abcam; ab109910). In this assay, complex IV was immunocaptured in the wells of an assay plate; its catalytic activity was determined colorimetrically based on the oxidation of reduced cytochrome *c*, and ELISA then measured its quantity in the same wells. The ratio of the activity and quantity represents the specific activity of complex IV. We clarify that this ratio of specific activity is referred to as “activity” throughout the manuscript. We lysed intact NDEVs in the assay buffer containing 10% detergent (provided in the assay kit) on ice for 30 min before loading the samples onto the assay plate. The plate was incubated overnight with gentle rocking at 4 °C followed by measurement of complex IV activity, quantity, and specific activity. All samples were run in duplicates. Using a commercial assay, we measured ATP synthase (Complex V) in NDEVs (abcam; ab109716). In this assay, ATP synthase was immunocaptured in the wells of an assay plate; its catalytic activity was determined colorimetrically based on the conversion of NADH to NAD^+^, and its quantity in the same sample wells was then measured by ELISA. The ratio of the activity and quantity represents the specific activity of ATP synthase.

Similarly, to complex IV, we use “activity” to describe this ratio throughout the manuscript. We lysed intact NDEVs in the assay buffer containing 10% detergent (both provided in the assay kit) before loading the samples onto the assay plate. The plate was incubated overnight with gentle rocking at 4 °C followed by measurement of ATP synthase activity, quantity, and specific activity. All samples were run in duplicates.

### 2.3. Bioenergetics Assessment

PBMCs preparation for bioenergetics analyses: Blood (5–7 mL) was collected in BD Vacutainer Cell Preparation Tubes^TM^ (Becton-Dickinson, Franklin Lakes, NJ, USA) according to the manufacturer’s recommendation within less than 1 h from blood collection. Most samples were collected between 9–11 a.m. Lymphocytes were isolated as previously described [37].

Chemicals and biochemicals: EDTA, EGTA, sodium succinate, mannitol, sucrose, and HEPES were all purchased from Sigma (St. Louis, MO, USA). Tris-HCl, glycine, sodium chloride, and potassium chloride were purchased from Fisher (Pittsburg, PA, USA). Bovine serum albumin (fatty-acid-free) was obtained from MP Biomedicals. All other reagents were of analytical or higher grade.

All samples were evaluated blindly and labeled with a 9-digit identification number, for which the cross-reference was available to those at the MIND Institute and not to the Giulivi team. The bioenergetic assessments followed essentially those described before [38,39]. For the polarographic determination of ATP-linked oxygen uptake of intact or permeabilized cells, we used a set-up of Clark-type oxygen electrodes with two chambers [12,39,40,41,42,43,44,45,46]. The semipermeable membranes were changed the day before the experiment was planned to avoid unwanted cell debris that may have become attached to it. The membrane was hydrated a day before (for no less than 8 h) to facilitate oxygen diffusion. Washes of the chamber were carried out with 70% ethanol and three washes of double-distilled, deionized water (18 megaohms). The calibration of the electrode entailed the recording of zero oxygen concentration (with dithionite) and air-saturate solution (used for functional studies) warmed up at 21–22 °C, at which the experiments were run. The calibration was run in duplicates with <10% CV. The oxygen concentration in the calibrating solution was calculated with the atmospheric pressure (barometer) and ambient temperature (thermometer). Additions to the chamber were carried out using Hamilton syringes to avoid increasing oxygen concentrations throughout the evaluations. The chamber was constantly stirred with a Teflon-coated minibar to ensure a homogenous diffusion of substrates and oxygen. Washes with 70% ethanol were warranted after using rotenone, antimycin, or FCCP, which tended to stick to the plastic walls of the chamber. ATP-driven oxygen uptake was usually carried out in duplicates at a given cell concentration (which was calculated before starting this protocol). All enzymatic assays were performed within the hour of collecting the blood sample and were run in parallel with controls. Reproducibility was ensured by running a subset of samples previously tested in parallel with new batches of samples.

Activities of complexes I–V in digitonin-permeabilized lymphocytes were determined by polarography essentially as described before [37,39]. Briefly, an aliquot (1.0–2.0 × 10^6^) of lymphocytes was added to the chamber equipped with a Clark-type Hansatech oxygen electrode at 20–22 °C in 0.3 mL of buffer containing 0.22 M sucrose, 50 mM KCl, 1 mM EDTA, 10 mM KH_2_PO_4_, and 10 mM HEPES, pH 7.4. Oxygen consumption rates were evaluated in air-saturated solutions in the presence of (i) 1 mM ADP plus 1 mM malate-10 mM glutamate followed by the addition of 5 μM rotenone; (ii) 10 mM succinate followed by the addition of 1 mM malonate; (iii) 1 mM α-glycerophosphate followed by the addition of 3.6 μM antimycin A; and (iv) 10 mM ascorbate and 0.2 mM *N*,*N*,*N*′,*N*′-tetramethyl-*p*-phenylenediamine followed by the addition of 1 mM KCN (activity of complex IV). Activities of individual electron transport chain (ETC) segments were evaluated as the difference in oxygen uptake recorded before and after the addition of specific inhibitors. Most mitochondrial inhibitors and uncouplers were stored at −80 °C as concentrated stock solutions (high mM) in DMSO to prevent unwanted oxidation or degradation. Quality control checks were performed with beef heart submitochondrial particles, and the results were compared to data collected over the years.

As previously described, oxygen consumption was also evaluated in intact cells using a Clark-type oxygen electrode (Hansatech, King’s Lynn, Norfolk, UK) [47,48]. ATP-linked oxygen uptake (or State-3-dependent oxygen uptake) was calculated as the difference between basal and oligomycin-induced State 4 oxygen uptake rates; State 4o is the residual respiration after the inhibition of ATP synthesis with the ATPase-specific inhibitor 0.2 µM oligomycin; maximal respiratory capacity, or State 3u, is described as the oxygen uptake rate in the presence of 2 µM of the uncoupler carbonyl cyanide-4-(trifluoromethoxy) phenylhydrazone (FCCP); the respiratory control ratio (RCR) was calculated as the ratio between States 3 and 4o; the index of respiratory capacity (IRC) was calculated as the difference between State 3 and State 4o normalized by that of State 3u. Mitochondrial proton leak (PL)/ROS production was calculated from the oligomycin-resistant oxygen consumption rates and normalized by basal respiration in the presence of 10 mM glucose-2 mM glutamine in RPMI-1640.

Citrate synthase activity was evaluated spectrophotometrically with a Tecan Infinite M200 microplate reader at 412 nm, as described before, using 2.5 to 3 × 10^5^ cells [37]. All cell pellets destined for this activity were tested within an hour following blood extraction. If stored, the pellets were supplemented with proteolytic inhibitors (4-benzenesulfonyl fluoride hydrochloride, EDTA, bestatin, E-64, leupeptin, and aprotinin, from Sigma) and kinase and phosphatase inhibitors (sodium orthovanadate, sodium molybdate, sodium tartrate, imidazole, cantharidin, (-)*p*-bromolevamisole oxalate, and calyculin A, from Sigma) and stored at −80 °C.

### 2.4. Statistical Analysis

Statistical analyses of the data were performed with the open-source R software, version 4.2.3. Results are expressed as mean ± standard deviation (SD) of the mean or median (25th percentile, 75th percentile) for continuous variables and frequency (%) for categorical variables. For quantitative variables, the normality of the data was assessed using Shapiro–Wilk’s test before statistical analysis. The change in quantitative variables pre- and post-treatment was analyzed using a paired *t*-test or Wilcoxon’s signed-rank test as appropriate. Spearman’s correlation analysis was conducted to calculate the magnitudes of correlation between a pair of quantitative variables and their significance. Linear regression was also used to describe relationships between two variables as appropriate. Two-tailed *p* < 0.05 values were considered statistically significant. The Benjamini–Hochberg false discovery rate (FDR) method was applied for bioenergetic data for multiple testing corrections, and FDR-adjusted *p*-values were suggested for guidance of significance when accounting for multiple testing given the small sample size of this pilot study with N = 11.

## 3. Results

### 3.1. Participants

A total of 15 participants aged 60–88 were enrolled from July 2021 to October 2022 Four participants did not complete the study; two were lost to follow-up, and two could not tolerate the starting dose. All participants reported no adverse effects. However, among the two individuals who could not tolerate the initial dose, they expressed complaints of heightened bloating, constipation, and diarrhea. Eleven participants (six males and five females) completed the study with data available both pre- and post-treatment, and their details are included in Table 1. The 11 participants were 60–88 years old at enrollment (median 74 years), with the *FMR1* premutation confirmed via molecular studies (mean CGG repeat size = 85) (Table 1). All participants were white and non-Hispanic.

### 3.2. Analysis of Outcome Measures

**Figure 2:**
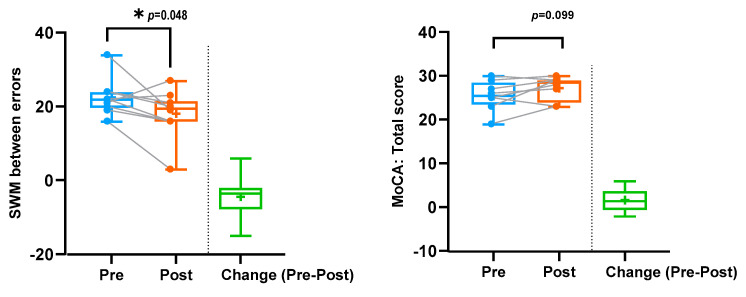
Efficacy of sulforaphane: changes in SWM between error and MoCA scores. Blue represents values/scores prior to sulforaphane treatment. Orange represents values/scores after sulforaphane treatment. Green represents the change in values/scores prior to and following sulforaphane treatment. * Significant at *p*-value < 0.05.

The changes in clinical outcome measures following the sulforaphane treatment were limited and are highlighted in Table 2 Interestingly, the spatial working memory score (SWM between errors) was significantly lower (improved) following the sulforaphane treatment (*p* = 0.048, FDR adjusted *p* = 0.43). After receiving the treatment, an upward trend was observed in the MoCA scores (*p* = 0.099, FDR adjusted *p* = 0.446). Figure 2 shows the changes from pre- to post-sulforaphane in SWM between the errors and MoCA scores.

**Figure 3:**
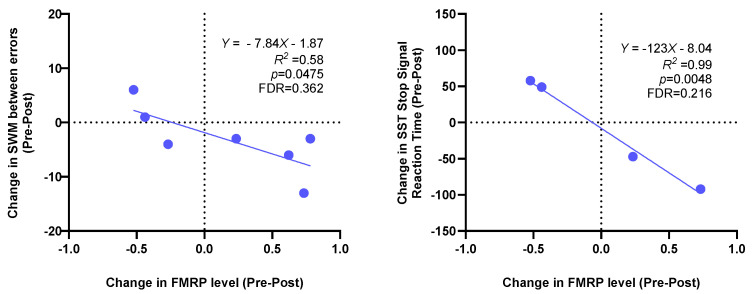
Correlations of post-sulforaphane changes in FMRP level with changes in SWM between errors and SST stop signal reaction time. FDR indicates an FDR-adjusted *p*-value.

**Figure 4:**
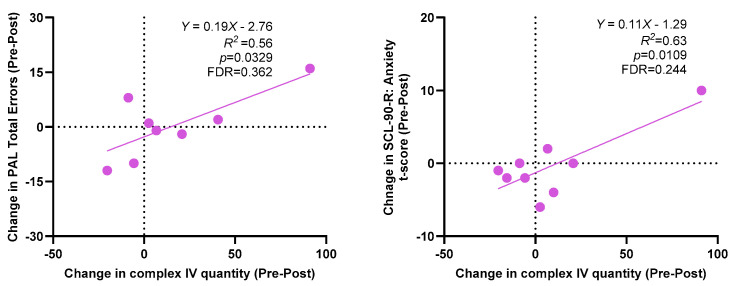
Correlations of post-sulforaphane changes in complex IV quantity in mitochondria-derived vesicles with changes in paired-associates learning (PAL) total errors and SCL-90-R anxiety scores. FDR indicates an FDR-adjusted *p*-value.

Table 3 presents the results of the molecular changes in mitochondria-derived vesicles following the sulforaphane treatment. On average, the post-treatment measurements indicated numerical increases in complex IV quantity, ATP synthase activity, and FMRP levels. However, it is important to note that these changes did not reach statistical significance. Figure 3 shows the relationships between increased FMRP levels and improvements in spatial working memory errors (SWM between errors) and stop reaction time (SST). Although these trends are visually presented, they did not achieve statistical significance after applying the false discovery rate (FDR) correction. Additionally, Figure 4 displays the correlation between the increased complex IV quantity levels and increased paired-associates learning (PAL) total errors as well as the SCL-90-R anxiety total scores. Once again, despite these observed correlations, they did not maintain statistical significance after the FDR correction.

### 3.3. Bioenergetics Results in PBMC

**Figure 5:**
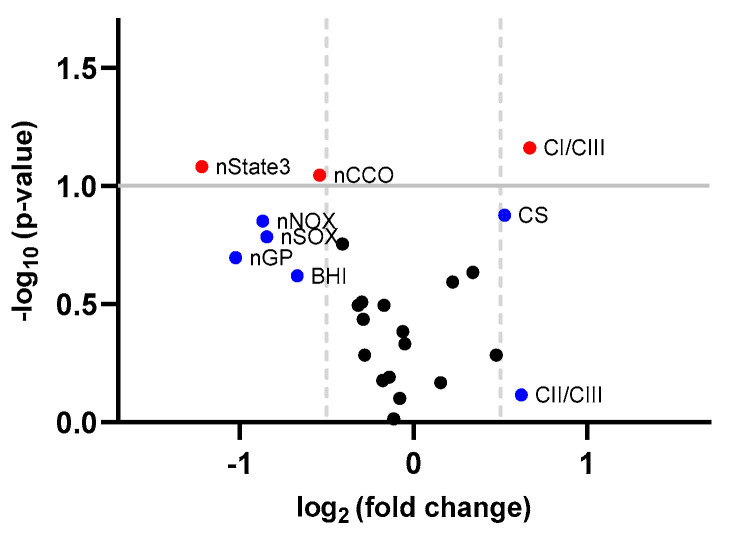
Volcano plot of bioenergetics in PBMC. Fold change (FC) represents the post-sulforaphane expression value relative to the pre-sulforaphane expression value. Red dots indicate bioenergetic measures with *p*-value < 0.1 and log_2_ (FC) > 0.5 or <−0.5, blue dots indicate bioenergetic measures with log_2_ (FC) > 0.5 or <−0.5 but *p*-value ≥ 0.1, and black dots are those within log_2_ (FC) < 0.5 or >−0.5 and *p*-value ≥ 0.1.

The changes in the bioenergetics from PBMC following sulforaphane treatment are summarized in Table 4. The volcano plot in Figure 5 displays the *p*-value versus the fold change for each bioenergetic measure in the post-sulforaphane expression value relative to the pre-sulforaphane expression value. On average, citrate synthase, a marker of mitochondrial mass, and the ratio of complex I to complex III were higher after the sulforaphane treatment. As a result of the higher activity of citrate synthase, the ATP production (with various substrates) normalized by this biomarker of mitochondrial mass was lower after the sulforaphane treatment (i.e., nState 3, nCCO, nNOX, nSOX, nGP). Notably, the higher ratio of complex I to complex III may indicate a decrease in complex III activity (as seen by the marginally higher ratio of complex II to complex III) accompanied by increases in complex I activity. This rearrangement of the complexes’ activities may indicate a more suitable management of NADH- vs. FADH_2_-linked substrates while minimizing the ROS production at complex III. Eight of the eleven subjects showed improvements (2 times the experimental error or log2FC of +/−0.3) in the ratio of complexes, seven in the normalized glycerophosphate-sustained ATP production, six in both citrate synthase activity and normalized malate-glutamate-sustained ATP production, and four in the malate-glutamate-sustained ATP production. Seven of the eleven subjects showed improvements in two or more outcomes (four with four outcomes and three with two to three outcomes), with the rest considered as not responsive to the treatment.

**Figure 6:**
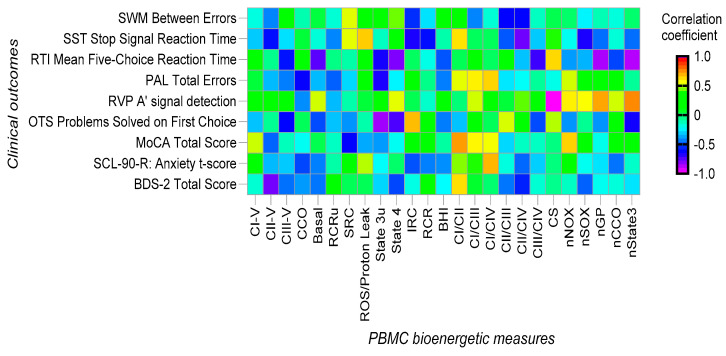
Heatmap of correlations between clinical measures and PBMC bioenergetic measures.

The correlation heatmap plot in Figure 6 shows the magnitudes of the correlations between the neuropsychiatric tests and PBMC bioenergetics (see Supplementary Table S1 for correlation coefficients and their *p*-values of significance). Three tests directly (positively) correlated with PBMC mitochondrial outcomes. The MoCA and BDS scores with the ratio of complex I to complex II and the SLC90-R anxiety t-score with the ratio of complex I to complex IV suggest the relevance of increasing complex I activity and indicating that a better management of NADH-linked substrates (such as glucose) improved these scores. The RVP A’ signal detection was positively correlated with the normalized (by citrate synthase activity) rate of ATP production sustained by glycerophosphate (nGP) and glucose (nState 3).

Four tests were negatively (indirectly) correlated with several PBMC mitochondrial outcomes. The RTI mean five-choice reaction time test was significantly negatively correlated with basal, State 4, CIII/CIV, nGP, and nState3, suggesting links to mitochondrial ROS production (i.e., the lower the mitochondrial ROS production, the higher the test scores). The BDS-2 total scores were inversely correlated with CII-V (correlation = −0.76; *p* = 0.007), suggesting that a better management of NADH-linked substrates via complex I (and not FADH_2_-linked via complex II) improved the ability of the patients to complete this test. The RVP A’ signal detection was negatively correlated with mitochondrial mass in PBMCs (citrate synthase biomarker (which explains the positive correlation with the normalized rates of ATP production sustained by glycerophosphate (nGP) and glucose (nState 3); see above)). The OTS problems solved on first choice scores were negatively correlated with State 3u, suggesting that more uncoupling (less ATP production) was associated with lower scores in this test.

None of the PBMC bioenergetic outcomes were significantly correlated with three of the six tests included under the CANTAB, namely SWM between errors, STT stop signal reaction time, and PAL total errors.

## 4. Discussion

Sulforaphane (SFN) is an isothiocyanate that stimulates antioxidant and cytoprotective effects by preventing the degradation of Nrf2 [49]. Nrf2, a leucine zipper (bZip) transcription factor, induces antioxidant/electrophile response elements (ARE), which are found in the promoter region of numerous genes involved in detoxification and cytoprotection [50,51]. We expected to see improvements in neuropsychiatric tests linked to measures of oxidative stress and mitochondrial function since in vitro studies of sulforaphane treatment have demonstrated this effect [21]. In this 6-month, open-label study of sulforaphane in 11 patients with FXTAS who completed this study, there was an improvement in spatial working memory (*p* = 0.048) compared to baseline. Additionally, increased FMRP levels in this study were correlated with improved working memory (fewer SWM errors) and decreased stop reaction time (SST). However, after the FDR adjustment, these were not significant. There were no statistically significant effects of sulforaphane in improving mitochondrial outcomes in mitochondria-derived vesicles, but there were near significant ones in PBMCs. While mitochondria-derived vesicles are not considered “functional” or to contribute to the cellular ATP budget, the bioenergetics of PBMCs showed improvements, mainly in the ratios of complex I to complex III and citrate synthase activity (mitochondrial mass).

Although doses of sulforaphane have shown limited efficacy in ASD [52] and in some cellular studies of neurodegenerative disorders [53,54,55], we did not expect sulforaphane to make a difference in the main features of FXTAS (tremor and ataxia), especially with the small cohort size and treatment window. Therefore, the improvement we found shows promise in terms of sulforaphane being a potential modulator of oxidative stress and neurocognitive features of FXTAS if studied in a larger population with a randomized controlled trial to better understand any efficacy.

While this study may be underpowered to reliably detect significant efficacy, it is worth noting that a positive trend in treatment effectiveness was observed with the MoCA scores, although it did not reach conventional statistical significance (*p* = 0.099). This is unusual for neurodegenerative diseases, as the MoCA score is known to be lowered by 3 points per year in those with Alzheimer’s disease (AD) [56]. A recent longitudinal study of MoCA scores in a cohort with major neurocognitive disorder/MCI decreased by 1.3 points over 1 year, which would equate to 0.6 points after 6 months [57]. Our cohort showed stability and some improvement in scores after 6 months. Despite this result not achieving significance, it suggests a significant improvement may be seen with a larger cohort, perhaps adjusting for normal decreases in scores with age in control cohorts or compared to other neurodegenerative diseases.

In the analysis of biomarkers, there was a non-significant trend toward an increase in the FMRP levels in PBMCs with treatment, an observation that would be consistent with evidence that Nrf-2 is a positive regulator of *FMR1* expression [58]; that is, stabilization of Nrf-2 with sulforaphane would be predicted to increase FMRP levels. When analyzing the increase in FMRP with clinical measures, we saw a significant positive correlation between FMRP levels and spatial working memory and signal stop time scores (Figure 3).

In terms of the bioenergetics of PBMCs, a substantial increase in the ratio of complex I to complex III activity and an improvement trend in two other outcomes (higher citrate synthase, lower complex III activity) were consistent with our previous study on FXTAS [21] and with other reports on neurodegenerative diseases [59,60] or other medical conditions [61,62,63]. The direct correlation of BDS2 with the ratio of complex I to complex III and the negative one with succinate-sustained ATP production underlines the link between NADH-generating substrates managed at complex I and improvements in executive function and dynamic motor behaviors. For the six tests included under CANTAB, only three (RTI, OTS, RVP A) showed correlations with PBMC bioenergetic outcomes. Taken together, the direct (with normalized rates of ATP production sustained by glucose and glycerophosphate) and indirect (with, e.g., citrate synthase, State 3u, complex III/complex IV, State 4) may point to minimizing uncoupling between electron transport and ATP production and mitochondrial ROS production. Thus, increasing mitochondrial mass and improving substrate management (NADH- vs. FADH_2_-linked substrates such as glucose vs. fat) while minimizing mitochondrial ROS production (and oxidative stress) seemed to improve some of the tests within CANTAB. Extending this conclusion, the BDS2 and MoCA scores were directly correlated with the ratios of complex I to complex II and negatively with the rates of ATP production that involved the primary use of FADH_2_-linked substrates via complex II (BDS only). Conversely, the SCL-90 test, which evaluates anxiety, was directly correlated with the ratios of complex I to complex IV. As no correlations were observed for this test and complex IV activity or complex-I-mediated ATP production, the implication of this finding in the context of anxiety is not clear.

While most subjects (seven of the eleven) showed some improvement in terms of PBMC bioenergetics, consistent with our previous study on the use of antioxidants [6], the lack of beneficial effects of this nutraceutical on all subjects can be understood by the concept of precision medicine, further exacerbated by the subject-dependent bioavailability of this compound, which is influenced by the microflora and diet [64].

### 4.1. Limitations

This study employed an open-label exploratory design, which lacks the controls of a randomized, double-blind trial. Additionally, the study utilized a relatively small sample size of patients, and it may not be representative of the broader FXTAS patient population. Since all the patients were white and non-Hispanic, this study does not represent ethnic and racial diversity, which we are now improving in current studies of FXTAS. The small and non-randomized sample size restricts the generalizability of the findings observed in this study and may not accurately represent the diversity of FXTAS patients in terms of age, disease severity, and other factors. While this study provides valuable preliminary insights and potentially hints at clinical significance for the future, its findings should warrant further investigation in larger, placebo-controlled clinical trials to establish the efficacy and safety of sulforaphane for FXTAS patients.

### 4.2. Sulforaphane in Neurogenerative Disorders

Sulforaphane has been shown to have neuroprotective effects through its influence on the NFEL2L pathway. In mice, the removal of NFEL2L or the combination of SFN with gamma-glutamylcysteine synthetase inhibitors resulted in the loss of the neuroprotective effects of SFN, indicating that SFN specifically targets NFEL2L. In addition to its neuroprotective effects, SFN also has anti-inflammatory properties. It can reduce inflammatory mediators such as TNF-α, and IL-6 and can decrease the activity of MAPKs like p38 and ERK 1/2. Sulforaphane can also lessen the cleavage of caspase-1 and caspase-3, which are involved in inflammation and apoptosis, and it can increase the release of anti-inflammatory cytokines like IL-4 and IL-10. Sulforaphane has also been found to promote autophagy in neurons through a mechanism that may involve NFEL2L or ERK and to improve mitochondrial function by activating genes that support mitochondrial biogenesis and preserve the production of ATP. Additionally, sulforaphane can enhance neurogenesis by increasing BDNF levels and activating the WNT signaling pathway [65].

A new compound was recently approved for treating Friedreich’s ataxia (FA), specifically omaveloxolone, which improves mitochondrial function, restores redox balance, and reduces inflammation. Omaveloxolone achieves this by activating NFEL2L. In a 48-week controlled trial of 150 mg per day in 103 patients with FA, there was a significant improvement in motor function as measured by the revised Friedreich ataxia rating scale [66]. This exciting breakthrough in treating a neurodegenerative disorder demonstrates the power of activating NFEL2L, so such trials should continue in FXTAS.

## 5. Conclusions

Our study offers valuable insights into the potential therapeutic benefits of sulforaphane for individuals afflicted by FXTAS. Although our open-label trial observed modest improvements in some clinical measures, these findings lend additional support to the promising role of sulforaphane in the treatment and management of FXTAS. This underscores the significance of ongoing research into sulforaphane as a therapeutic agent and its potential to enhance cognitive and mitochondrial outcomes. Further investigations are warranted to validate and refine these initial results and to explore the broader potential of sulforaphane and other NFEL2L activators in addressing the challenges posed by FXTAS.

## Figures and Tables

**Table 1:** Descriptive statistics of baseline characteristics of patients completed the trial (N = 11).

Variable	N	Mean (SD) or Frequency (%)	Median (Q1, Q3)	Range
Age (years)	11	73.47 (8.95)	74.37 (67.33, 78.2)	60.73–88.66
Gender	11			
Male		6 (54.5%)		
Female		5 (45.5%)		
FXTAS diagnosis	11			
Definite		6 (54.5%)		
Probable		3 (27.3%)		
Possible		2 (18.2%)		
FXTAS stage	11	3.05 (0.79)	3 (2.5, 3.75)	2–4
2		3 (27.3%)		
3		4 (36.4%)		
3.5		1 (9.1%)		
4		3 (27.3%)		
Age of onset: tremor	10	59.25 (7.31)	58.5 (53.38, 64.5)	49–73
Age of onset: ataxia	8	66.38 (5.93)	66 (62.75, 70)	57–75
AGG interruptions	11	1.09 (0.54)	1 (1, 1)	0–2
0		1 (9.1%)		
1		8 (72.7%)		
2		2 (18.2%)		
CGG repeats	11	85.27 (13.16)	87 (72, 92.5)	70–110
Activation ratio (in females)	5	0.54 (0.14)	0.49 (0.46, 0.53)	0.44–0.78
*FMR1* mRNA level	11	2.50 (0.34)	2.57 (2.19, 2.77)	1.99–2.94

**Table 2:** Efficacy of sulforaphane: changes in clinical outcomes following sulforaphane treatment.

Variable	N	Pre-Sulforaphane	Post-Sulforaphane	Change from Baseline (Pre-Post)	Cohen’s *d* Effect Size	*p*-Value *	FDR #
		Mean (SD)	Mean (SD)	Mean (SD)			
CANTAB							
SWM between errors	10	22.6 (4.74)	18.2 (6.37)	−4.4 (6.08)	−0.72	0.048 ^1^	0.43
SST Stop Signal Reaction Time	7	303.7 (43.75)	283.58 (59.67)	−20.11 (58.21)	−0.35	0.396 ^1^	0.713
RTI Mean Five-Choice Reaction Time	10	417.22 (68.97)	436.07 (89.78)	18.85 (73.97)	0.25	1 ^2^	1
PAL Total Errors	8	16.88 (6.71)	17.12 (7.75)	0.25 (9.05)	0.03	0.94 ^1^	1
RVP A’ signal detection	8	0.87 (0.04)	0.89 (0.03)	0.02 (0.03)	0.67	0.159 ^1^	0.476
OTS Problems Solved on First Choice	7	8.71 (3.2)	7.43 (3.95)	−1.29 (2.98)	−0.43	0.298^1^	0.67
MoCA total score	8	25.5 (3.46)	27.25 (2.76)	1.75 (2.6)	0.67	0.099 ^1^	0.446
SCL-90-R: Anxiety t-score	9	57.78 (9)	57.44 (8.22)	−0.33 (4.53)	−0.07	0.831 ^1^	1
BDS-II total score	11	23.36 (2.38)	23.27 (2)	−0.09 (3.14)	−0.03	0.926 ^1^	1

* *p*-value was obtained by paired *t*-test ^1^ or Wilcoxon’s signed-rank test ^2^ to test whether the mean or median of the pre-sulforaphane scores was significantly different than the mean or median of their post-sulforaphane scores, respectively. # FDR represents an adjusted *p*-value calculated by the Benjamini–Hochberg FDR procedure in multiple hypothesis testing to control the expected proportion of false discoveries.

**Table 3:** Efficacy of sulforaphane: molecular changes in mitochondria-derived vesicles following sulforaphane treatment.

Variable	N	Pre-Sulforaphane	Post-Sulforaphane	Change from Baseline (Pre-Post)	Cohen’s *d* Effect Size	*p*-Value *	FDR #
		Mean (SD)	Mean (SD)	Mean (SD)			
Complex IV quantity	11	29.2 (17.46)	39.77 (35.14)	10.57 (31.78)	0.33	0.52 ^2^	0.866
Complex IV specific activity	11	0.45 (0.2)	0.44 (0.32)	−0.01 (0.34)	−0.03	0.918 ^1^	0.918
ATP synthase quantity	11	35.81 (11.98)	41.8 (17.35)	5.99 (15.05)	0.40	0.216 ^1^	0.866
ATP synthase specific activity	11	0.00225 (0.00128)	0.00262 (0.00246)	0.00037 (0.00251)	0.15	0.765 ^2^	0.918
FMRP level	7	1.03 (0.445)	1.193 (0.715)	0.162 (0.569)	0.28	0.478 ^1^	0.866

* *p*-value was obtained by paired *t*-test ^1^ or Wilcoxon’s signed-rank test ^2^ to test whether the mean or median of the pre-sulforaphane scores is significantly different than the mean or median of their post-sulforaphane scores, respectively. # FDR represents an adjusted *p*-value calculated by the Benjamini–Hochberg FDR procedure used in multiple hypothesis testing to control the expected proportion of false discoveries.

**Table 4:** Efficacy of sulforaphane: changes in bioenergetic measures in PBMC following sulforaphane treatment.

Variable	N	Pre-Sulforaphane	Post-Sulforaphane	Change from Baseline (Pre-Post)	log_2_ (Fold Change)	*p*-Value *	FDR
		Mean (SD)	Mean (SD)	Mean (SD)			
CI-V	11	0.358 (0.197)	0.453 (0.206)	0.095 (0.248)	0.34	0.232 ^1^	0.572
CII-V	11	0.455 (0.275)	0.507 (0.351)	0.052 (0.403)	0.16	0.678 ^1^	0.771
CIII-V	11	0.42 (0.393)	0.388 (0.325)	−0.032 (0.379)	−0.11	0.966 ^2^	0.966
CCO	11	0.998 (1.301)	0.887 (0.481)	−0.11 (1.015)	−0.17	0.32 ^2^	0.572
Basal	11	0.686 (0.52)	0.558 (0.362)	−0.128 (0.397)	−0.30	0.31 ^1^	0.572
RCRu	11	1.651 (0.813)	1.35 (0.733)	−0.301 (1.054)	−0.29	0.366 ^1^	0.61
SRC	11	1.908 (1.257)	1.828 (1.335)	−0.08 (1.493)	−0.06	0.413 ^2^	0.645
ROS/proton leak	11	1.327 (1.054)	1.281 (0.391)	−0.046 (0.789)	−0.05	0.465 ^2^	0.684
State 3u	11	1.04 (0.571)	0.834 (0.542)	−0.206 (0.653)	−0.32	0.319 ^1^	0.572
State 4	11	0.715 (0.413)	0.676 (0.437)	−0.039 (0.47)	−0.08	0.79 ^1^	0.823
IRC	11	0.651 (0.234)	0.761 (0.35)	0.11 (0.303)	0.23	0.255 ^1^	0.572
RCR	11	1.046 (0.617)	0.86 (0.238)	−0.186 (0.583)	−0.28	0.52 ^2^	0.684
BHI	11	1.067 (0.75)	0.671 (0.46)	−0.396 (0.661)	−0.67	0.24 ^2^	0.572
CI/CII	11	1.054 (0.82)	1.466 (1.426)	0.412 (1.695)	0.48	0.52 ^2^	0.684
CI/CIII	11	1.435 (1.261)	2.28 (1.789)	0.846 (1.377)	0.67	0.069 ^1^	0.572
CI/CIV	11	0.818 (0.881)	0.742 (0.78)	−0.076 (0.528)	−0.14	0.643 ^1^	0.771
CII/CIII	11	1.431 (0.667)	2.199 (2.233)	0.768 (2.412)	0.62	0.765 ^2^	0.823
CII/CIV	11	0.772 (0.727)	0.682 (0.391)	−0.09 (0.672)	−0.18	0.666 ^1^	0.771
CIII/CIV	11	0.692 (0.624)	0.521 (0.415)	−0.171 (0.391)	−0.41	0.176 ^1^	0.572
CS	11	2.003 (1.458)	2.879 (1.115)	0.876 (1.777)	0.52	0.133 ^1^	0.572
nNOX	11	0.336 (0.346)	0.184 (0.109)	−0.152 (0.315)	−0.87	0.141 ^1^	0.572
nSOX	11	0.352 (0.325)	0.196 (0.134)	−0.156 (0.344)	−0.84	0.164 ^1^	0.572
nGP	11	0.356 (0.4)	0.175 (0.242)	−0.181 (0.437)	−1.02	0.201 ^1^	0.572
nCCO	11	0.56 (0.397)	0.385 (0.326)	−0.175 (0.308)	−0.54	0.09 ^1^	0.572
nState3	11	0.582 (0.677)	0.25 (0.298)	−0.332 (0.64)	−1.22	0.083 ^1^	0.572

* *p*-value was obtained by paired *t*-test ^1^ or Wilcoxon’s signed-rank test ^2^ to test whether the mean or median of the pre-sulforaphane scores were significantly different compared to the mean or median of their post-sulforaphane scores, respectively. * FDR represents an adjusted *p*-value calculated by the Benjamini–Hochberg FDR procedure used in multiple hypothesis testing to control the expected proportion of false discoveries.

## Data Availability

Data are contained within the article and Supplementary Materials.

## Supplementary Materials

The following supporting information can be downloaded at: https://www.mdpi.com/article/10.3390/cells12242773/s1, Table S1. Spearman’s correlation coefficients (corresponding *p*-values of significance) between changes in clinical and PBMC bioenergetic measures. * FDR represents an adjusted *p*-value calculated by the Benjamini–Hochberg FDR procedure used in multiple hypothesis testing to control the expected proportion of false discoveries.
