# Supplementary material for: Open-Label Sulforaphane Trial in FMR1 Premutation Carriers with Fragile-X-Associated Tremor and Ataxia Syndrome (FXTAS)
Source: Cells. 2023 Dec 5;12(24):2773. doi: 10.3390/cells12242773 (PMC10741398; doi:10.3390/cells12242773)
Supplement: Supplementary file 1 [file cells-12-02773-s001.zip › cells-2633387-supplementary.pdf]

Open-Label Sulforaphane Trial in FMR1 Premutation Carriers with Fragile-X-Associated Tremor and Ataxia Syndrome (FXTAS)

Ellery Santos [1,2,*], Courtney Clark [2], Hazel Maridith B. Biag [1,2], Si Jie Tang [2], Kyoungmi Kim [1,3], Matthew D. Ponzini [1,3], Andrea Schneider [1,2], Cecilia Giulivi [1,4], Federica Alice Maria Montanaro [5,6], Jesse Tran-Emilia Gipe [4], Jacquelyn Dayton [4], Jamie L. Randol [7], Pamela J. Yao [8], Apostolos Manolopoulos [8], Dimitrios Kapogiannis [8], Ye Hyun Hwang [7], Paul Hagerman [1,7], Randi Hagerman [1,2] and Flora Tassone [1,7]

1 Medical Investigation of Neurodevelopmental Disorders (MIND) Institute, University of California Davis Health, Sacramento, CA 95817, USA; hbbiag@ucdavis.edu (H.M.B.B.); kmkim@ucdavis.edu (K.K.); mdponzini@ucdavis.edu (M.D.P.); anschneider@ucdavis.edu (A.S.); cgiulivi@ucdavis.edu (C.G.); pjhagerman@ucdavis.edu (P.H.); rjhagerman@ucdavis.edu (R.H.); ftassone@ucdavis.edu (F.T.)
2 Department of Pediatrics, School of Medicine, Sacramento, University of California, Davis, CA 95817, USA
3 Division of Biostatistics, Department of Public Health Sciences, University of California, Davis, CA 95616, USA; cjcclark@ucdavis.edu (C.C.); sijtang@ucdavis.edu (S.J.T.)
4 Department of Molecular Biosciences, School of Veterinary Medicine, University of California, Davis, CA 95616, USA; jegipe@ucdavis.edu (J.T.-E.G.); jrdayton@ucdavis.edu (J.D.)
5 Child and Adolescent Neuropsychiatry Unit, Department of Neuroscience, Bambino Gesù Children's Hospital, IRCCS, 00165 Rome, Italy; federica.montanaro@opbg.net
6 Department of Education, Psychology, Communication, University of Bari Aldo Moro, 70121 Bari, Italy
7 Department of Biochemistry and Molecular Medicine, School of Medicine, University of California, Davis, CA 95616, USA; jlrandol@ucdavis.edu (J.L.R.); yehhwang@ucdavis.edu (Y.H.H.)
8 Laboratory of Clinical Investigation, Intramural Research Program, National Institute on Aging, National Institutes of Health, Baltimore, MD 212241, USA; yaopa@grc.nia.nih.gov (P.J.Y.); apostolos.manolopoulos@nih.gov (A.M.); kapogiannisd@mail.nih.gov (D.K.)
* Correspondence: ersantos@ucdavis.edu; Tel.: +1-916-703-0200

Supplementary Table S1. Spearman's correlation coefficients (corresponding p-values of significance) between changes in clinical and PBMC bioenergetic measures.

	SWM Between Errors	SST Stop Signal Reaction Time	RTI Mean Five-Choice Reaction Time	PAL Total Errors	RVP A' signal detection	OTS Problems Solved on First Choice	MoCA Total Score	SCL-90-R: Anxiety t-score	BDS-2 Total Score
(NOX) CI-V	-0.15 (0.671)	-0.29 (0.556)	0.19 (0.608)	0.07 (0.882)	0.17 (0.703)	-0.3 (0.518)	0.54 (0.168)	0.36 (0.339)	-0.13 (0.697)
(SOX) CII-V	-0.39 (0.260)	-0.57 (0.200)	-0.03 (0.946)	-0.31 (0.462)	0.24 (0.582)	-0.04 (0.937)	-0.44 (0.272)	-0.31 (0.415)	-0.76 (0.007)
(GP) CIII-V	0.33 (0.358)	-0.25 (0.595)	-0.56 (0.096)	-0.4 (0.327)	0.12 (0.793)	-0.59 (0.161)	-0.1 (0.821)	-0.29 (0.456)	-0.43 (0.192)

CCO	-0.08 (0.826)	0.07 (0.906)	0.19 (0.608)	-0.62 (0.115)	-0.38 (0.360)	0.04 (0.937)	-0.18 (0.670)	-0.49 (0.183)	-0.36 (0.275)
Basal	0.07 (0.853)	-0.14 (0.783)	-0.72 (0.024)	-0.31 (0.462)	0.55 (0.171)	-0.48 (0.274)	0.05 (0.910)	-0.4 (0.282)	-0.42 (0.203)
RCRu	-0.14 (0.709)	-0.39 (0.396)	-0.02 (0.973)	-0.45 (0.267)	-0.31 (0.462)	-0.3 (0.518)	-0.11 (0.799)	-0.07 (0.864)	0.25 (0.464)
SRC	0.57 (0.088)	0.57 (0.200)	-0.07 (0.865)	-0.26 (0.536)	-0.07 (0.882)	-0.37 (0.413)	-0.6 (0.117)	0.36 (0.339)	0.04 (0.904)
ROS/Proton Leak	0.25 (0.493)	0.68 (0.110)	0.15 (0.682)	0.12 (0.793)	0.07 (0.882)	-0.07 (0.875)	-0.34 (0.417)	0.52 (0.150)	0.04 (0.904)
State 3u	0.34 (0.329)	-0.11 (0.840)	-0.64 (0.054)	-0.64 (0.096)	0.31 (0.462)	-0.82 (0.025)	-0.36 (0.382)	-0.19 (0.618)	-0.27 (0.430)
State 4	0.46 (0.179)	0.14 (0.783)	-0.77 (0.014)	-0.21 (0.619)	0.57 (0.151)	-0.7 (0.077)	-0.18 (0.670)	0.02 (0.966)	-0.49 (0.126)
IRC	-0.52 (0.121)	-0.64 (0.139)	0.03 (0.946)	0.26 (0.536)	0.05 (0.935)	0.7 (0.077)	0.13 (0.756)	-0.47 (0.201)	-0.16 (0.638)
RCR	-0.25 (0.493)	-0.57 (0.200)	-0.16 (0.657)	-0.02 (0.977)	-0.12 (0.793)	0.15 (0.751)	0.37 (0.365)	-0.4 (0.282)	0.23 (0.490)
BHI	0.14 (0.696)	-0.07 (0.906)	-0.47 (0.178)	-0.4 (0.327)	0.17 (0.703)	-0.41 (0.364)	-0.06 (0.888)	-0.38 (0.316)	-0.2 (0.553)
CI/CII	0.3 (0.397)	0.64 (0.139)	0.04 (0.919)	0.57 (0.151)	0 (1.000)	-0.3 (0.518)	0.75 (0.031)	0.48 (0.192)	0.65 (0.030)
CI/CIII	-0.39 (0.260)	0.04 (0.963)	0.21 (0.560)	0.62 (0.115)	0.55 (0.171)	0.11 (0.812)	0.61 (0.108)	0.43 (0.250)	0.08 (0.810)
CI/CIV	-0.14 (0.709)	0.04 (0.963)	0.03 (0.946)	0.67 (0.083)	0.38 (0.360)	0.04 (0.937)	0.59 (0.126)	0.71 (0.031)	0.01 (0.979)
CII/CIII	-0.6 (0.068)	-0.46 (0.302)	0.39 (0.263)	-0.24 (0.582)	0.07 (0.882)	0.56 (0.195)	-0.26 (0.528)	-0.15 (0.698)	-0.42 (0.203)
CII/CIV	-0.6 (0.068)	-0.75 (0.066)	-0.3 (0.407)	-0.19 (0.665)	0.45 (0.267)	0.11 (0.812)	-0.11 (0.799)	-0.44 (0.240)	-0.54 (0.083)
CIII/CIV	-0.1 (0.774)	-0.29 (0.556)	-0.68 (0.035)	-0.05 (0.935)	0.4 (0.327)	-0.48 (0.274)	-0.11 (0.799)	-0.28 (0.470)	-0.13 (0.697)
CS	0.05 (0.893)	0.43 (0.354)	0.66 (0.044)	-0.17 (0.703)	-0.95 (0.001)	0.52 (0.233)	-0.01 (0.978)	-0.01 (0.983)	-0.03 (0.925)
nNOX	0.02 (0.960)	-0.18 (0.713)	-0.36 (0.313)	0.55 (0.171)	0.62 (0.115)	-0.37 (0.413)	0.67 (0.069)	0.31 (0.415)	-0.11 (0.748)
nSOX	-0.22 (0.550)	-0.64 (0.139)	-0.32 (0.368)	0.17 (0.703)	0.6 (0.132)	-0.04 (0.937)	0.22 (0.608)	-0.18 (0.650)	-0.45 (0.166)
nGP	-0.09 (0.813)	-0.43 (0.354)	-0.83 (0.006)	0.21 (0.619)	0.74 (0.046)	-0.37 (0.413)	-0.17 (0.691)	-0.24 (0.527)	-0.18 (0.600)
nCCO	-0.15 (0.684)	-0.14 (0.783)	-0.49 (0.154)	0.24 (0.582)	0.55 (0.171)	0.15 (0.751)	0.29 (0.490)	-0.42 (0.260)	-0.15 (0.667)
nState3	0.01 (0.973)	-0.43 (0.354)	-0.84 (0.004)	-0.05 (0.935)	0.79 (0.028)	-0.63 (0.129)	0.19 (0.649)	-0.12 (0.763)	-0.24 (0.472)
